# Employment Decisions of Newly Graduated Occupational Therapists

**DOI:** 10.1177/00084174241274742

**Published:** 2024-09-09

**Authors:** Stephany Lui, Jeffrey Boniface, Giovanna Boniface, Donna Drynan

**Keywords:** Employment choice, Extrinsic factors, Fieldwork, Intrinsic factors, New graduates, Surveys, Workforce planning, Choix de l'emploi, enquêtes, facteurs extrinsèques, facteurs intrinsèques, nouveaux diplômés, planification de la main-d'œuvre, travail sur le terrain

## Abstract

**Introduction.** Employment decision-making is essential for understanding workforce trends. Current occupational therapy workforce research describes distribution disparities of occupational therapists within geographic locations, services such as acute care or community health, and private or public sectors. New graduates of occupational therapy programs are critical to meeting the demand and distribution disparities of occupational therapy services in British Columbia. However, recent employment decision-making of new occupational therapy graduates has not been well studied. **Purpose.** This study aimed to examine factors that influence newly graduated occupational therapists’ employment decisions. **Methods.** This descriptive study sampled 122 occupational therapists who were registered in one province and graduated from a Canadian occupational therapy program between 2017 and 2022. Data was collected through an online survey about intrinsic factors, extrinsic factors, and past fieldwork experiences that affected participants’ employment decision-making. Descriptive data analysis was used to organize participants’ responses. **Findings.** Results identified that work-life balance and mentorship were the highest rated factors that influenced participants’ current and first employment respectively. Participants agreed that the variety and number of placements they had as students were more influential to their employment decisions than the length of the fieldwork education. **Conclusion.** This study identified the intrinsic and extrinsic factors in employment choices that may influence recruitment, retention, and workplace planning of new graduates.

## Résumé

**Description**. La prise de décision en matière d'emploi est essentielle pour comprendre les tendances de la main-d'œuvre. Les recherches actuelles sur la main-d'œuvre en ergothérapie décrivent des disparités dans la répartition des ergothérapeutes entre les zones géographiques, les services tels que les soins aigus ou la santé communautaire, et les secteurs privé et public. Les personnes nouvellement diplômées dans des programmes d'ergothérapie sont essentielles pour répondre à la demande et combler les disparités dans la distribution des services d'ergothérapie en Colombie-Britannique. Cependant, la prise de décision en matière d'emploi des personnes nouvellement diplômées en ergothérapie ces dernières années n'a pas été bien étudiée. **But**. Cette étude examine les facteurs qui influencent les décisions d'emploi des personnes nouvellement diplômées en ergothérapie. **Méthodologie**. À partir d'un échantillon de 122 ergothérapeutes ayant obtenu l'agrément dans une province canadienne et reçu leur diplôme entre 2017 et 2022, des données ont été recueillies au moyen d'un sondage en ligne axé sur les facteurs intrinsèques, les facteurs extrinsèques et les expériences antérieures de stage ayant influencé leur prise de décision en matière d'emploi. Une analyse descriptive des données a été utilisée pour organiser les réponses des personnes participantes. **Résultats**. L'analyse descriptive a révélé que l'équilibre travail-vie professionnelle et le mentorat étaient les principaux facteurs d'influence pour les choix d'emploi actuels et initiaux, respectivement. En outre, les personnes participantes ont souligné l'importance de la variété et du nombre de stages pendant leurs années d'études par rapport à la durée des stages. **Implications**. Ces résultats soulignent l'importance de comprendre les facteurs intrinsèques et extrinsèques dans les décisions d'emploi, ce qui peut éclairer les stratégies de recrutement, de maintien en poste et de planification de la main-d'œuvre pour les personnes nouvellement diplômées.

## Introduction

Occupational therapists are dedicated to working alongside people of all ages with various health conditions and they are essential in the promotion of overall well-being and engagement in meaningful activities (WFOT, 2023). According to the World Health Organization (2024), 2.4 billion people in the world experience health conditions that would benefit from rehabilitation services. The shortage of occupational therapists has been a major challenge in meeting the global demand of rehabilitation services (Richards & Vallée, 2020). In Canada, occupational therapists are integral to the healthcare system. Data suggests that occupational therapists are expected to face labour shortages over the period of 2019–2028 (Occupational Therapist (OT) in Canada | Job Prospects, n.d.). For British Columbia (BC), the shortage of occupational therapists has been discussed for many years. The supply of occupational therapists has been disproportionate with population growth (CAOT-BC, 2015). Population estimates from BC Statistics indicate rather a growth from 4,628,500 to 6,118,300 by 2041, an estimated increase of 32% (CAOT-BC, 2015). The demand for occupational therapy services is projected to grow continuously in the coming years due to various factors such as aging population, increased life expectancy, technological advancements, greater social health awareness, and a shift towards ambulatory care (CAOT-BC, 2015).

The shortage of occupational therapists has been reported internationally as well. Ballmer et al. (2023) inform of a disparity in the occupational therapy profession related to diversity, geography, and area of practice. Understanding employment decisions is important for workforce planning. In BC, the largest source of supply to the occupational therapy workforce each year are graduates from the occupational therapy programs in Canada. In 2020, 86.2% of the supply of occupational therapists in BC were graduates from a Canadian program (CIHI, 2020). Research on employment decision-making has been conducted with various healthcare student populations such as medical students, nurses, and physiotherapists (Gottlieb-Smith et al., 2021; Hall et al., 2021; Sud et al., 2020; Wilkinson et al., 2016) and influential factors included lifestyle, personal interest, and clinical placement experiences. However, the factors that affect employment decisions vary across different healthcare professions and have yet to be studied in newly graduated occupational therapists. The employment decision processes of other healthcare providers are often investigated through examining the intrinsic and extrinsic factors involved in the decision (Balmer et al., 2020; Campbell et al., 2012). Intrinsic and extrinsic factors were originally known as motivation and hygiene factors from Herzberg's two-factor theory (Herzberg, 1993). Intrinsic factors, also called motivation factors, are personal needs coming from within the individual that relates to growth and self-actualization. Extrinsic factors, also known as hygiene factors, come from outside the individual and are related to the conditions surrounding the job. Intrinsic factors and extrinsic factors exist in an inverse relationship that is governed by employees’ expectations. When expectations for intrinsic factors are met, they contribute to job satisfaction. When expectations for extrinsic factors are not met, they lead to job dissatisfaction, but if they do, they can prevent job dissatisfaction.

Further research has suggested that intrinsic characteristics such as self-perceived skills and competencies can also contribute to employment choices and the success of entry level occupational therapists in practice (Holmes & Scaffa, 2009; Millsteed et al., 2017). In addition to intrinsic and extrinsic factors, fieldwork education experiences have been shown to affect students’ future practice preferences (Chiang et al., 2013; Crowe & Mackenzie, 2002; Sloggett et al., 2003). Exposure to related work helps students prepare for future employment (Rodger et al., 2007). Newly graduated occupational therapists are more willing to look for future employment in the area of their fieldwork education experience (Benaroya et al., 2021). To better understand future occupational therapy workforce supply in BC, additional data are needed on employment decision-making processes. This descriptive study aims to fill this information gap on new graduate occupational therapy employment preferences. The purpose of the study is to examine the intrinsic and extrinsic factors, and fieldwork education experiences that influence occupational therapists’ employment decisions in BC.

## Method

### Study Design

This study utilized an online survey to collect quantitative description of employment patterns of the newly graduated occupational therapist population in BC and factors affecting their employment decision making. Approval was obtained from the University Behavioural Research Ethics Board (BREB number: H22-02041).

### Participants and Recruitment

There is no clear definition of how many years post graduation an individual is to be considered as newly graduated. This study attempted to draw a line between new graduates and other occupational therapist by their effectiveness to problem solve and apply the occupational therapy process with the situation. In other words, their professional judgement and reasoning. Schell (2018) suggested that differing levels of occupational therapy professional reasoning are obtained as early as five years into practice. Based on this timeline, occupational therapists registered to practice in BC, who graduated from an accredited occupational therapy program in Canada within the last 5 years (2017–2022) were recruited to participate in the study using this timeline.

The recruitment of participants was achieved with the assistance of College of Occupational Therapists Of British Columbia (COTBC). COTBC provided contact information of registrants who consented to receive research-related information during registration. Consent was implied if participants initiated the anonymous online survey; no self-identifying indicators were collected.

### Data Collection

The survey was drafted through literature review and feedback from three occupational therapy researchers who are part of the research team. To ensure survey technical soundness, the survey was completed by one occupational therapist fitting the criteria of the study, who provided feedback that increases clarity of the survey. The anonymous online survey was distributed to the 631 occupational therapists on the list provided by the regulatory college. Data was backed up and stored on a secure Canadian server in compliance with the Freedom of Information and Protection of Privacy Act (FIPPA).

The 20-min online survey contained 19 questions with skip logic function. This allowed the participants to progress through the survey based on their answer to the question “is your current employment different from your first employment?” Therefore, not all participants answered all questions. The closed questions comprised of multiple choice, Likert scales and matrix table formats which collected information on (a) the time required to, and the priority of obtaining employment after graduation; (b) current employment, such as practice area, region, sector, employment status, and intrinsic and extrinsic factors; (c) first employment if applicable, such as practice area, region, sector, employment status, and intrinsic and extrinsic factors; (d) fieldwork education experiences such as sector, number of fieldwork education, area of practice, educator to student ratio, and influence of fieldwork on employment decision; and (e) demographics, such as age, age of graduation, and years of practice. In cases where participants’ current employment is different from their first employment, they were directed to answer questions regarding the characteristics of their first employment, and to rate the intrinsic and extrinsic factors that affect employment. The survey was designed as such to observe any differences in ratings of factors related to employment decisions.

The list of intrinsic and extrinsic factors used in this study was derived with reference to Herzberg (1993) based on the first and second researcher's knowledge of employment decision, and reviewed by the other three occupational therapy researchers of the research team. A 7-point Likert scale was chosen for evaluation of fieldwork education experiences as it is more likely to capture participant's true subjective assessment of the statements given that the survey was distributed electronically (Finstad, 2010).

The online survey was open for seven weeks (Leeuw et al., 2008). A total of four emails were sent to the participants. The initial email was sent on November 7, 2022. To increase response rate, follow-up emails were sent with reminders and changes to the email messages at week one, three, seven, and eight (Ruel et al., 2016).

### Data Analysis

The data were analyzed using IBM Statistics Package for the Social Sciences (SPSS) version 26.0 to produce descriptive statistics. Findings were reported in percentages, means, and standard deviations.

## Results

Six-hundred thirty-one occupational therapists were invited to participate in the study. In total, 169 people responded to this survey (overall response rate 27%). Twenty-seven responses were excluded as they were incomplete. The final study sample consisted of 122 occupational therapists for an overall response rate of 19%.

As presented in Table 1, participants were mostly cis-women (86.1%), aged less than 30 years old (64.8%) who had been practicing as occupational therapists for 1–2 years (44.3%). Almost all participants reported finding employment was their top priority upon graduation (95.9%) and 60.7% found employment before graduation.

**Table 1:** Characteristics of Participants

Characteristics	*n*	%
Age, years		
≤ 30	79	64.8
31–40	41	33.6
41–50	2	1.6
Gender		
Cis-woman	105	86.1
Cis-man	14	11.5
Non-binary	1	0.8
Prefer to self-identify	2	1.6
Age of graduation, years		
22–30	97	79.5
31–38	23	18.9
38–45	2	1.6
Is finding employment a priority upon graduation?		
Yes	117	95.9
No	15	4.1
Time needed to find employment upon graduation, months		
Before graduation	47	60.7
< 1	23	18.9
2–3	19	15.6
4–6	5	4.1
7 ≥	1	0.8
Years of practise		
1-2	54	44.3
2-3	21	17.2
3-4	12	9.8
4 >	31	25.4

*Note*. Percentage reported by cases.

### Employment Profile

Approximately 49.2% of participants’ employment upon graduation was different from current employment. Participants were asked to rate the satisfaction with their current employment on the scale of 1–5, with 1 being extremely satisfied and 5 being extremely dissatisfied. Most participants reported that they were “satisfied” with their current employment (54.1%). Spearman's correlation was conducted to investigate the relationship between participants’ ratings of intrinsic and extrinsic factors and their level of job satisfaction. There was a significant negative relationship between sense of challenge and level of job satisfaction, *r*_s_([*N*(122)−2]) = −.206, *p *= .023. Participants are more likely to be more satisfied with their job if sense of challenge is an important factor that affects their employment decisions.

Table 2 shows the characteristics of participant's employments, including employment status, region, and area of practice. Patterns of employment were similar for both current and first employment. Most participants worked in the public sector (current employment: 63.9%, first employment: 75%). Participants’ employments were mostly located in the major cities of the province (current employment: 50%, first employment: 51.7%). General physical health was the most common area of practice (current employment: 61.5%, first employment: 61.7%).

**Table 2:** Characteristics of Participants’ Employment

Characteristics	First employment^a^		Current employment^b^	
	*n*	%	*n*	%
Employment status				
Public: Full-time	21	35.0	53	43.4
Public: Part-time	6	10.0	11	9.0
Public: Temporary	4	6.7	4	3.3
Public: Casual	14	23.3	10	8.2
Private: Full-time	11	18.3	35	28.7
Private: Part-time	4	6.7	7	5.7
Combination of both public and private sector	3	5	10	6.8
Non-practising	—	—	1	0.8
Self-employed	—	—	5	4.1
Contracted	2	3.3	9	7.4
Others	1	1.7	2	1.6
Employment region				
Metro Vancouver	31	51.7	61	50.0
Vancouver Island & Gulf Islands	11	18.3	29	23.8
Thompson Okanagan	3	5.0	9	7.4
Fraser Valley	11	18.3	19	15.6
Northern BC	3	5.0	8	6.6
Kootenay Rockies	3	5.0	4	3.3
Sunshine Coast/Whistler	1	1.7	3	2.5
Area of practice				
General physical health	37	61.7	75	61.5
Mental Health	24	40.0	53	43.4
Neurological	23	38.3	52	42.6
Musculoskeletal	27	45.0	53	43.4
Vocational rehabilitation	8	13.3	29	23.8
Client service management	1	1.7	12	9.8
Medical/legal	4	6.7	5	4.1
Health promotion and wellness	3	5.0	12	9.8
Teaching	—	—	4	3.3
Research	1	1.7	3	2.5
Cardiovascular/respiratory	5	8.3	11	9.0
Palliative care	13	21.7	17	13.9
Digestive/metabolic endocrine	1	1.7	1	0.8
Other areas of direct service provision	6	10.0	6	4.9
Other areas of practice	14	23.3	33	27.0

Note: Participants were allowed to select all that apply or multiple responses.

^a^ *n* = 60, participants were only asked to provide details of their first employment if current employment is different from their first.

^b^ *N* = 122.

### Factors Influencing Employment Decision

Participants were given a table with a list of eight intrinsic factors and 16 extrinsic factors. They were then asked to rate the importance of each factor on the scale of 1–5 (with 5 being extremely important and 1 being not important at all) in their decision for finding employment, results are outlined in Table 3.

**Table 3:** Factors Influencing Employment Decisions

Factors		First employment^a^		Current employment^b^	
		*M*	*SD*	*M*	*SD*
Intrinsic					
	Family and/or partner commitments	3.30	1.28	3.54	1.22
	Lifestyle/work-life balance	3.67	1.02	4.53	0.58
	Passion/setting you are interested in	3.42	1.17	4.26	0.77
	Sense of accomplishment	3.40	0.94	4.01	0.61
	Personality/fit	3.52	0.91	4.34	0.65
	Self-efficacy (competency)	3.58	0.79	4.00	0.69
	Sense of control over own work	3.13	0.89	4.03	0.77
	Sense of challenge	3.12	0.90	3.81	0.79
Extrinsic					
	Work hours	4.00	0.94	4.35	0.67
	Job security	3.95	0.95	4.29	0.83
	Provision of mentorship	4.38	0.90	4.31	0.80
	Prestige/status	2.37	0.94	2.39	0.98
	Job autonomy/ Flexibility	3.18	1.05	3.92	0.89
	Income/salary	3.48	0.89	4.13	0.70
	Availability of job	4.17	0.81	3.87	0.74
	Healthcare/pension benefits	3.62	1.11	3.84	1.01
	Company policy and administration	2.97	0.92	3.38	0.85
	Interpersonal relations (colleagues, supervisor etc.)	3.70	0.93	4.19	0.75
	Location of work	3.82	1.00	4.11	0.76
	Skill/professional development	4.00	0.76	4.11	0.64
	Job advancement (career growth)	3.37	0.82	3.66	0.77
	Recognition (from employer, clients etc.)	3.08	0.85	3.27	0.94
	Reputation of facility	2.97	0.94	3.38	0.95
	Appearance of facility	2.63	0.96	2.84	1.06

^a^ *n* = 60. Participants were only asked to rate the importance of factors that influence employment decision if their current employment is different from their first employment.

^b^ *N* = 122.

The three most highly rated factors that influenced participants’ current employment were a mix of intrinsic and extrinsic factors, they were: (a) lifestyle/work-life balance, (b) work hours, (c) personality/fit to the job. However, the three most highly rated factors that influenced participants’ first employment were solely extrinsic, they were: (a) provision of mentorship, (b) availability of job, (c) skills/professional development offered by the job.

Participants were asked to rate three statements about the influence of characteristics of fieldwork education to their employment decision. They were given eight options: from strongly agree to strongly disagree in addition to a “not applicable” option. Most participants “disagreed” (30.3%) that the length of fieldwork affected their employment decision. Most respondents “agreed” that the number of fieldwork (36.1%) and different areas of practice they had in their fieldwork (41.8%) affected their employment decision.

## Discussion

Occupational therapists are currently facing labour shortages in Canada (Occupational Therapist (OT) in Canada | Job Prospects, n.d.). The supply of occupational therapists is expected to be disproportionate with the estimated 32% population growth in BC by 2041 (CAOT-BC, 2015). Participants’ employment profiles aligned with the statistics of the profile of BC occupational therapists in 2022 (College of Occupational Therapists of British Columbia, 2022), which showcased that majority of BC occupational therapists worked in the major city in the province and practiced in general physical health and in the public sector. Although participants tended to work in the above employment profile, the results of this study suggested that newly graduated occupational therapists tend to change jobs early in their career. Participants were inclined to prioritize provision of mentorship in their first employment, but valued lifestyle/work-life balance more in their current employment.

This study highlights the importance of mentorship for newly graduated occupational therapists. Participants prioritize mentorship opportunities in their first employment, which aligns with existing research that highlights the challenges of first year practice. New graduates often describe their first year of practice as overwhelming, and they appreciate feedback and support from more experienced occupational therapists (Hodgetts et al., 2007; Seah et al., 2011). Effective mentorship programs, as suggested by Fitzgerald et al. (2015) and Moores and Fitzgerald (2017), should consist of supervision, support, and education to foster clinical reasoning, professional identity, active learning, and reflective practice. This equips newly graduated occupational therapists with essential skills to manage caseloads, develop clinical skills and knowledge, participate and contribute actively within their professional community, and gain confidence to practice (Turpin et al., 2021).

Access to mentorship programs, and the gap between desired and actual mentorship experiences, appeared to affect participants’ employment decisions. The perceived lack of structured mentorship programs in rural settings, as highlighted by Devine (2006), might explain participant's preference to work in urban settings. This underscores the need for tailored mentorship programs that reflects the varied needs of community population and considers factors like geographic location and workplace structure. Formalized mentorship programs would ensure reliable support for new graduates. Furthermore, research supports that mentorship programs are associated with higher retention rates and job satisfaction (Falzarano & Zipp, 2012). Feeling valued through mentorship likely contribute to these factors, ultimately benefiting both workplaces and newly graduated occupational therapists.

Work-life balance emerged as the most influential factor in participants’ current employment decisions, similar to other healthcare providers like nurses and doctors (Picton, 2021; Price et al., 2018). This focus on work-life balance is likely due to the demanding nature of these professions. Occupational therapists face cognitively and emotionally demanding tasks, with workload being a major predictor of exhaustion that they face in daily practice (Gupta et al., 2012; Teo et al., 2021). Maintaining appropriate separation between work and personal lives contributes to job satisfaction by mitigating the emotional exhaustion that occupational therapists experience in the workplace. This is particularly important in BC's public healthcare system (HSA, 2021), as significant staffing shortages exists across health disciplines. Conversely, private practice may offer higher perceived job autonomy and greater flexibility that are associated with lower burnout rates (Scanlan et al., 2013). However, recent research suggests that private practice comes with its own challenge. The lack of mentorship and self-employment within a competitive market, especially early in an occupational therapist's career can lead to higher rates of burnout in private practice (Bruce et al., 2022). These stressors, along with consideration for work-life balance, could account for newly graduated occupational therapists’ preference to work in the public sector.

Interestingly, this study also identified a correlation between job satisfaction and the importance of sense of challenge in participants’ employment decision. Participants who rated sense of challenge important tend to report higher job satisfaction, which aligns with literature that suggests cognitive challenges of work as a key predictor of job satisfaction (Judge et al., 2023). According to Hackman and Oldham's (1980) Job Characteristics Model, the sense of challenge is impacted by the importance of the task, the variety of skills that are required in the task, the degree of autonomy the employee has, the feedback that employees receive regarding their performance, and employee's ability to see their work through from the beginning to the end. This suggests that creating a work environment that respects and recognizes the value of occupational therapy, promote professional growth and development for newly graduated occupational therapists, alongside initiatives that promote work-life balance, could be crucial for attracting and retaining new graduates.

The observed shift in priorities from mentorship in participants’ first employment to work-life balance in their current roles, aligns with previous research that suggests attaining work-life balance is a dynamic, ongoing process involving the re-evaluation of goals and priorities (Raja & Stein, 2014). Early in their careers, new graduates’ desire for career success often led them to focus on work over their personal lives (Sturges & Guest, 2004). It appears that newly graduated occupational therapists are likely to prioritize learning and consolidation of clinical skills in the beginning of their careers. Once their learning needs were met, they seem to begin to re-evaluate their professional and personal priorities, which could explain a shift to consider work-life balance in their current employment decisions. This shift in priorities, along with the emotionally and cognitively demanding nature of occupational therapy work, suggests the need for a multifaceted approach when addressing occupational therapist workforce planning.

In fact, the process of evaluating work-life balance can be impacted by supports that the employee receives on the micro, meso, and macro levels (Abendroth & Dulk, 2011). Supports can consist of employees’ friends and family providing help within the domestic sphere, supervisors and colleagues that are understanding of the employees’ situation, and workplace policies such as flexible working arrangement as well as public policies on provincial and federal levels that influence the structure of workplaces and personal lives. Therefore, it is important to consider workplace policies and the supports that occupational therapists receive and when considering workforce planning.

Fieldwork education experiences, a key component of occupational therapy education, plays an important role in shaping new graduate's clinical preferences (Christie et al., 1985). Participants confirmed this, mostly agreed that the number and variety of fieldwork education experiences affected their employment decision. This aligns with Caron (2000) who suggested that fieldwork education helped students identify and clarify preference for practice areas, and the variety of fieldwork education offered an opportunity for the development of familiarity in differing areas of practice. Interestingly, this study showed that less participants tend to work in private practice, potentially due to fewer number of private practice fieldwork education opportunities compared to the public sector. Kobbero et al. (2018) indicated that only 3% of occupational therapists had past fieldwork experiences in private practice. Participants’ preference to work in general physical health and in the public sector suggested that there are higher number of fieldwork education available in those settings. Students are unlikely to enter a clinical area in which they had no fieldwork experience in, and are more likely to be employed in the practice settings in which they had the most fieldwork experiences (Crowe & Mackenzie, 2002). It appears that the number of fieldwork education opportunities in multiple sectors and practice settings are important considerations the anticipated growth of occupational therapy demand in BC.

### Study Limitations

This study has several limitations. First, the descriptive design of the study restricts data to be analyzed to display statistical associations between the factors and employment patterns. Second, the survey assumed universal understanding of the list of intrinsic and extrinsic factors. Without defining each intrinsic factor and extrinsic factor, participants response might vary depending on how they interpret each factor. Third, generalizability of the findings is limited by the small sample size and low response rate. Most of the participants identified as having one to two years of experience, potentially excluding perspectives of other newly graduates with more practice experience and higher workloads. The findings are also specific to BC and cannot be generalized to all newly graduated occupational therapists in Canada. Additionally, survey distribution during a holiday season could affect response rate as it might coincide with other priorities that participants have during that time.

### Future Research

Future research could employ an exploratory design to establish the statistical association between factors and employment decisions. This would provide a more robust understanding of the specific factors that are relative to each aspect of newly graduated occupational therapists’ employment patterns. Employment preferences can be analyzed with detail statistics of available jobs to further understand workforce distribution in BC. Given how participants prioritize mentorship in their first employment, future research could also explore effective ways to apply mentorship programs to ensure both the successful transition to practice and potentially increase retention rates. This study also identified work-life balance as participants’ priority for their current employment and importance of sense of challenge to their job satisfaction. Further research can explore how occupational therapists conceptualize work-life balance and sense of challenge in their work. This would allow organizations to gain a better understanding of how sense of challenge could be fostered and how work-life balance can best be supported to enhance job satisfaction and increase retention rates.

This study can be replicated in later years as the number of fieldwork education in private practice is rising (Drynan, 2023). It can also be replicated on a national scale to examine the interjurisdictional differences, as well as different health authority's configurations to newly graduated occupational therapists’ employment decision-making process. Information of such would be beneficial for future occupational therapy workforce planning to ensure the equitable delivery of occupational therapy services across sectors and settings, while maintaining desirable work-life balance within the profession.

## Conclusion

This descriptive study provides the most up to date knowledge of newly graduated occupational therapists’ employment decision and highlights the importance of a multifaceted approach to occupational workforce planning in BC. The results of this study indicated that new graduates tend to work in the major city in the province and, in general physical health and in the public sector. Newly graduated occupational therapists tend to prioritize mentorship in the beginning of their career, then shift to prioritize work-life balance in their current roles. Addressing the need for mentorship, creating a work environment that promotes positive sense of challenge, and initiative to promote work-life balance appears to be important for attracting newly graduated occupational therapists. In addition, ensuring a variety of fieldwork education across different areas of practice could also broaden their job choices. Given the shortages of occupational therapists in BC, these results provide insight of the potential changes that could be made regarding recruitment and retention of occupational therapists.

## Key Messages

- Formal mentorship programs should be considered as a recruitment strategy for newly graduated occupational therapists in BC entering the workforce.
- Work-life balance policies should be considered when drafting recruitment and retention strategies for occupational therapists.
- Fostering a sense of challenge in a workplace could increase job satisfaction of newly graduated occupational therapists.
